# Effects of Prenatal Exposure to Aflatoxin B1: A Review

**DOI:** 10.3390/molecules26237312

**Published:** 2021-12-02

**Authors:** João Victor Batista da Silva, Carlos Augusto Fernandes de Oliveira, Leandra Náira Zambelli Ramalho

**Affiliations:** 1 Department of Pathology and Forensic Medicine, School of Medicine of Ribeirão Preto, University of São Paulo, Ribeirao Preto 14049-900, Sao Paulo, Brazil; joaovsilva@usp.br (J.V.B.d.S.); lramalho@fmrp.usp.br (L.N.Z.R.); 2 Department of Food Engineering, School of Animal Science and Food Engineering, University of São Paulo, Pirassununga CEP 13635-900, Sao Paulo, Brazil

**Keywords:** aflatoxins, carcinogenicity, mutagenicity, prenatal exposure, teratogenicity

## Abstract

Aflatoxins are mycotoxins produced as secondary fungal metabolites. Among them, aflatoxin B1 (AFB1) stands out due to its genotoxic and mutagenic potential, being a potent initiator of carcinogenesis. In this review, the outcomes from the published literature in the past 10 years on the effects of AFB1 pathophysiological mechanisms on embryological and fetal development are discussed. In several animal species, including humans, AFB1 has a teratogenic effect_,_ resulting in bone malformations, visceral anomalies, lesions in several organs, and behavioral and reproductive changes, in addition to low birth weight. The mutagenic capacity of AFB1 in prenatal life is greater than in adults, indicating that when exposure occurs in the womb, the risk of the development of neoplasms is higher. Studies conducted in humans indicate that the exposure to this mycotoxin during pregnancy is associated with low birth weight, decreased head circumference, and DNA hypermethylation. However, as the actual impacts on humans are still unclear, the importance of this issue cannot be overemphasized and studies on the matter are essential.

## 1. Introduction

Mycotoxins are secondary products of fungal metabolism, causing harmful effects on human and animal health. These substances are commonly found in food, especially when harvest storage or transport practices are inadequate. It is estimated that about 25% of all food worldwide is contaminated with mycotoxins, but some studies suggest that this is an underestimation [1]. As acute intoxications are less common in humans, the effects of chronic exposure to mycotoxins have been more extensively studied and seem to be related to a wide range of health disorders [2]. Among mycotoxins, aflatoxins stand out due to their carcinogenic potential. Aflatoxins (AFs) are produced mainly by Aspergillus spp. fungi and are found in several foods, such as corn, peanuts, and others. Although not all food contaminated by fungi have AFs, these substances occur all over the world, with temperature and humidity providing adequate conditions for contamination by Aspergillus spp. and the production of these mycotoxins [3].

Some AFs are produced directly by Aspergillus, whilst others are the result of the metabolism of these substances in the liver after intake. The four main types of aflatoxins found in foods are variants B1, B2, G1, and G2, with aflatoxin B1 (AFB1) having the highest carcinogenic potential. Aflatoxin M1 (AFM1) is a product of aflatoxin B1 metabolism in the animal organism, and stands out both for its carcinogenic potential and for being excreted in the milk of animals and humans [4]. AFB1 is known to have genotoxic, mutagenic, immunogenic, and hepatotoxic potential, and for causing acute liver damage when ingested in large quantities. It also has a remarkable teratogenic potential, and several studies were carried out on its effects during the prenatal life of animals and humans, especially on fetal development.

Knowing that this mycotoxin has the ability to cross the placental barrier and has already been identified in human umbilical cord samples [5,6], the main objective of this review is to discuss the effects, already reported in the scientific literature, of the pathophysiological mechanisms of AFB1 on embryological and fetal development. For this purpose, articles published in the last 20 years reporting experiments carried out in mice, rats and rabbits were selected, as well as articles on human exposure in the prenatal period and the association between exposure and impacts on health of fetuses and newborns. For this, the ISI Web of Knowledge, PubMed, Google Scholar, and Scopus databases were accessed, and the following terms were searched: ‘‘Aflatoxin’’, ‘‘prenatal exposure’’, ‘‘human’’, and ‘‘teratogenicity’’.

## 2. Aflatoxin B1 Biotransformation and Mechanism of Action

**Figure 1:**
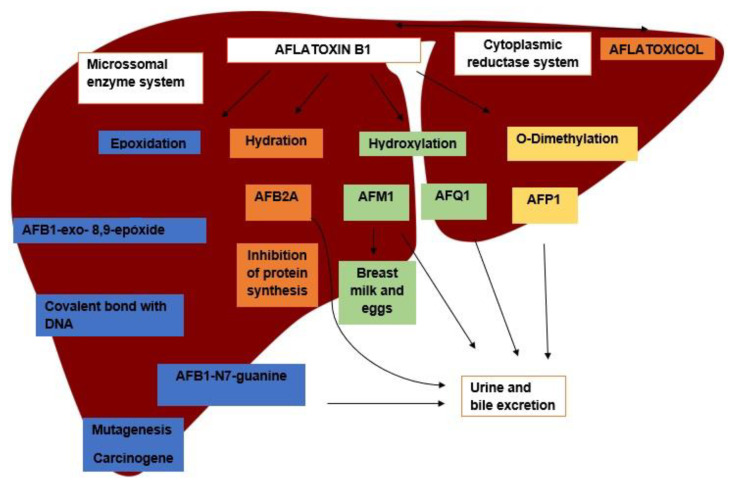
Main metabolization pathways of aflatoxin B1 in the liver, its metabolites, and excretion pathways.

After ingestion, AFB1 is rapidly absorbed from the intestine and reaches the liver to be metabolized by mixed-function oxidases. AFB1 goes through a complex process of biotransformation; in the first stage, it may go through different metabolization pathways, yielding several metabolites, followed by the conjugation process for excretion (Figure 1). AFB1 may also go through a reversible reduction process in the cytoplasmic reductase system of hepatocytes, yielding to aflatoxicol (AFL), which can be transformed again into AFB1, becoming a source of its own storage. Metabolization of AFB1 culminates with the formation of several metabolites, e.g., aflatoxin P1 (AFP1) and aflatoxin Q1 (AFQ1) [7].

Among the metabolization pathways of AFB1, the epoxidation process stands out. In this process, AFB1 is converted into AFB1-8.9-epoxide (AFBO), which is able to bind to macromolecules, such as those of DNA, RNA, and proteins, forming adducts responsible for the toxic potential of the aflatoxins [8]. The binding of AFBO to guanine in the DNA molecules results in the formation of AFB1-N7-Guanine (AFGuan), which is excreted in urine. The production of AFGuan leads to a guanine to thymine substitution in the third base of codon 249 of the host DNA, which is the main basis of the carcinogenic effect of AFB1, as this mutation is responsible for the loss of function of the p53 gene, a trigger for hepatocellular carcinoma (HCC) [9].

Another important route of AFB1 metabolization is hydration, forming AFM1. AFM1 can also go through an epoxidation process and become AFM1-epoxide, which has the same ability to bind to macromolecules as AFBO [10]. AFB1 and AFM1 are classified by the IARC (International Agency for Research on Cancer), respectively, as group I carcinogen and group 2B carcinogen, respectively [11,12]. In addition, AFM1 can be found in human and animal milk, becoming a unique health issue, as animal exposure to AFB1 makes humans vulnerable [13,14,15]. Finally, hydration also yields AFB2A, an important inhibitor of protein synthesis that is related to the effects of acute intoxication after high AFB1 intake [8,16].

## 3. Effects of Prenatal Exposure to Aflatoxin b1 in Animals

Several harmful effects are associated with exposure to AFB1 during the prenatal period, such as low birth weight, small litters, fetal death and resorption, bone and visceral deformities, reproductive changes, impact on immune capacity, and behavioral changes, in addition to a predisposition to neoplasm development [17].

### 3.1. Bone Malformations

Bone defects related to intrauterine exposure to AFB1 are the most common problems reported in the literature. In experiments, they are evidenced in several species of animals. Bone defects are most commonly related to ossification failures, changes in bone size and shape, and the absence or alteration of some bone accidents (Table 1).

When the effects on bone development are compared, the three studies in Table 1 demonstrated similar effects. However, it is worth noting the difference in the doses used by Abdulrazzaq et al. [18] (20 mg/kg), which is high compared to the other studies, but used as a single dose, either on the 7th or on the 13th day. El Nahla et al. [19] administered the lowest dose (0.05 mg/kg/day) to rabbits, by gavage, between the 6th and the 18th day of gestation. Fetaih et al. [20] administered 1 mg/kg/day to rats. The similarity of effects on bone formation found in the study by Abdulrazzaq et al. [18] and the other studies may be due to the high dose used, which would explain the similarity, even when exposure was not constant. However, when routes of administration are compared, peritoneal administration [20] is less representative of real contamination by AFB1, which mostly occurs by oral route. The study by Abdulrazzaq et al. [18] also showed that a single dose in the pre-implantation period impacted uterine growth and may have played a role in the failure of fetal development. This is an issue to be clarified in future studies. Another important point that should be considered in further studies is the variation in the susceptibility to AFB1 between species. For example, among the animals evaluated in these studies, rabbits were more sensitive with LD50 of 0.3 mg/kg, while mice and female rats were more resistant, with LD50 of 9.0 and 17.9 mg/kg, respectively [21].

The mechanisms involved in the failure of fetal development are not well elucidated. It is suggested that AFB1 may affect the transcription of genes associated with bone development, impacting processes such as intramembrane mineralization and endochondral ossification [19,20]. The results found in these studies are in agreement with the effects reported in previous ones, confirming the impact of prenatal AFB1 exposure in bone development [22,23,24]. Regarding the moment of exposure, animals exposed during embryonic development seem to be at increased risk of minor malformations, such as those affecting bone accidents [18].

### 3.2. Visceral Changes

Several visceral changes have been reported, among them, the decrease in size and weight of the liver and kidneys [18,19]. Regarding histopathological findings, the liver shows more significant changes, with the presence of fatty degeneration, congestion, and necrosis. Although these changes may also be found in the kidneys, they are less intense (Table 2). Other histological alterations are evidenced in the organs of the reproductive tract (discussed below).

Table 2 shows the hepatotoxic potential of AFB1 in the prenatal period, a finding reported in all the studies cited. The most common changes involved degenerative and congestive lesions. Supriya and Reddy [26] found decreased fetal liver weight when 20 μg/kg of AFB1 was injected intramuscularly in rats from the 12th to the 19th day of gestation, showing the effect of this toxin on the liver of the fetus, even in low doses. However, it should be noted that the intramuscular route was used, which does not represent the natural route of exposure to this toxin and disregards aspects involved with the digestive process, which should be investigated in future studies.

El-Nahla et al. [19] are the only authors citing cardiac changes in fetuses, with decreased heart size and ventricular lumen. Changes were evidenced in rabbit fetuses even when exposed to low doses (0.05 mg/kg), as this species is intensely susceptible to the harmful effects of AFB1 [21]. The studies by El-Nahla et al. [19] and Fetaih et al. [20] found similar effects on kidneys, mostly degenerative changes. Finding these changes, even in different species (rabbits and rats), led to the hypothesis that the kidney may be the second most impacted viscera in prenatal exposure to AFB1. Thymus alterations found by Fetaih et al. [20] also support another hypothesis: exposure to AFB1 may have an immunotoxic effect in the prenatal period, just like in adults, favoring the onset of infectious diseases and/or facilitating the development of neoplastic processes [27].

Supriya and Reddy [26] also demonstrated that a dose of 10 μg/kg of AFB1 in rats leads to changes in the testicular morphology of the fetus, which worsens with increasing doses. This finding indicates that this mycotoxin has a potent effect on the reproductive function of animals exposed in the prenatal period. Future investigations should be carried out on alterations in testicles and ovaries using exposure by gavage to evaluate deleterious effects caused by exposure to AFB1, especially in production animals.

Mechanisms of injury and visceral changes occur by increased oxidative stress, leading to structural injury caused by lipid peroxidation, lesions to other macromolecules, and reduced protein synthesis. The liver is always more affected because it is the main site of action of AFB1. The evident renal alterations are possibly due to the function of the kidneys as blood filters, predisposing this organ to toxic injury. In addition, together with bile, kidneys are the main sources of excretion of AFB1 and its metabolites. Therefore, its tubules are also susceptible to toxic injury [7,17,20,28].

### 3.3. Reproductive Changes

Exposure to AFB1 can result in several reproductive alterations, especially in males, with impacts on reproductive behavior, sperm production, and testicular and epididymal morphology. However, the most significant finding involves serum hormone levels, marked by a decrease in testosterone [29,30,31]. When exposure occurs in the prenatal period, the effects seem to be even more remarkable. Supriya et al. [30] found a significant decrease in testosterone levels in male rats exposed to 10, 20, and 50 μg/kg of AFB1 during embryonic development. The same study showed increased levels of luteinizing hormone (LH) and follicle stimulant hormone (FSH). Other reproductive alterations were also found, such as decreased sperm volume and viability, as well as morphological alterations in testicles and the tail of the epididymis.

The mechanisms through which reproductive damage occurs are still being elucidated. AFB1 may act as a potential endocrine disruptor, interfering with the hypothalamus–hypophysis–testicular axis, leading to hormonal dysfunction. The consequences of hormonal disruption may be more severe when exposure occurs in the embryonic phase [26,32]. Another possibility would be the ability of AFB1 to bind to the acute steroidogenic regulatory protein (STAR), thus affecting the transfer of cholesterol to the mitochondria, which has a negative impact on steroidogenesis [30]. Direct cellular damage caused by oxidative stress should also be considered, as already evidenced by Althnaian et al. [33], who found substantial increases in oxidative stress markers and decreases in antioxidant enzymes in the testicles of rats exposed to a single intraperitoneal application of 3 mg/kg of AFB1 [33]. AFB1 also affects the reproductive capacity of females, although in lower intensity, causing follicular atresia [32]. However, no studies were found to evidence this effect when exposure occurs before birth.

### 3.4. Genotoxicity and Mutagenicity

AFB1 presents high genotoxicity, which is probably its most overwhelming effect, notably leading to several chromosomal aberrations in animals exposed to it [34]. Both DNA damage and mutations can result from the injury caused by AFB1 metabolites (AFBO), and by the oxidative stress that results from the metabolization of this mycotoxin. In the fetuses of rats exposed to 1 mg/kg of AFB1 between the 6th and the 15th day of gestation, several chromosomal aberrations in bone marrow cells were evidenced, mainly gap and breakage lesions, indicators of the genotoxic potential of this mycotoxin in the prenatal period [20].

The mechanisms by which AFB1 causes DNA damage in fetal life seem to be similar to those affecting adult animals, by the metabolization of AFB1 into AFBO in the liver of the fetus and the identification of AFGuan adducts in fetuses [35]. The apurinic site left by AFGuan (after it is released and excreted) is filled by a thymine base, which characterizes the main mutation caused by AFB1, the G:C → T:A transversion in the P53 gene. This change may initiate the carcinogenesis process. These transversions are also evidenced in the fetuses of rats exposed to 6 mg/kg of AFB1 by peritoneal application in a single dose on the 14th day of gestation [17,36].

Another metabolite found in rat fetuses exposed to AFB1 is AFB1-Fapy. This metabolite is the result of a chemical transformation in the aflatoxin molecule, resulting in the opening of the furan ring, and creating a more stable molecule that remains linked longer to DNA. AFB1-Fapy is considered to be more carcinogenic because it prevents repair enzymes from being activated, as it produces less evident structural damage to the DNA helix [7,35,36]. Chawanthayatham et al. [36] also showed that fetuses present about 1% of adducts found in mothers. However, when the mutations are identified, the difference is 4.6%. These numbers indicate that the ability of AFB1 to cause mutation is 20 times greater during prenatal exposure than in adults.

These phenomena are explained by the fact that the fetal liver has the capacity to metabolize AFB1 into AFBO, but has a decreased ability to excrete AFB1 metabolites due to the low number of conjugate enzymes of Phase II of metabolization (e.g., glutathione transferase). Moreover, during fetal life, liver cells are in constant multiplication, which may lead to the expansion of mutations [35,36]. Another important mechanism of genotoxicity is the oxidative stress caused by the metabolization of AFB1, leading to the formation of several adducts that act remotely, causing neoplasms at a distance [7]. Animals exposed to AFB1 have a decrease in the amount of antioxidant enzymes and an increase in markers of oxidative stress [17,20]. Thus, treatment with antioxidant agents may provide protection against oxidative stress and, consequently, reduce the carcinogenic potential of these substances [37,38]. Finally, the importance of epigenetic lesions, mainly DNA methylation and modifications in histones, cannot be discarded in genotoxicity and mutagenicity processes [39].

### 3.5. Other Changes

Other important changes are low birth weight, both in rats and rabbits, besides the decrease in the number of offspring per litter [18,19,20,25,26]. In rabbits, 0.05 mg/kg/day AFB1 from the 6th to the 18th day leads to increased size of the orbits, microphthalmia, wrinkled skin, and eyelids with fewer hair follicles [19].

Behavioral changes in terms of locomotion and reflex were reported in rats treated with 20 and 50 μg AFB1/kg, from the 12th to the 19th day of gestation [26]. Rats treated with 1 mg/kg from the 6th to the 15th day of gestation presented adactyly and exophthalmia [25].

### 3.6. Other Animals

As demonstrated in the studies on mammals, birds seem to be susceptible to the effects of aflatoxin during embryonic development, resulting in malformations, mainly in the early stages of development, as demonstrated by Veselý et al. in chicken embryos [40]. Other studies also demonstrate that these animals can be impacted by immune deficiency, defects in the development of the bursa of fabricius, and even death before hatching, which can represent great economic loss [41]. The deleterious effects of AFB1 are not restricted to birds and mammals, and it is possible to observe neurological changes in zebrafish when exposed to this mycotoxin at a dose of 15–75 ng/mL 6 h after fertilization, with marked behavioral change [42]. Additionally, in zebrafish embryos there are failures in the embryonic development of the liver due to marked apoptosis, in addition to immunological deficiencies [43]. In general, aflatoxin similarly impacts morpho development during the prenatal period, in addition to impacting mainly the liver of several animal species. Another issue to be considered is the susceptibility of the species to the effects of mycotoxins, with birds being more sensitive, while fish show a wide variation in sensitivity [21].

Moreover, when it comes to hepatic metabolism, the physiological variation between species is an important challenge to be overcome. The findings in experiments with different species provide indications that AFB1 has the potential to have its effects increased, especially in embryos and fetuses, but more studies are needed to elucidate the safe dose for consumption in this critical period [36]. Therefore, standardizing the form of exposure and gestation period in the experiments would be a way to clarify still uncertain points about prenatal exposure to AFB1, thus enabling us to reach more knowledge about the real impacts of this substance in the human prenatal period. This is only possible with an exposure assessment, by measuring biomarkers in vulnerable populations and monitoring the health of newborns and children in places with high exposure to AFB1.

## 4. Prenatal Exposure in Humans

Most unfavorable birth outcomes, such as premature births, miscarriages, low birth weight, and even stillbirths, occur in developing countries [44]. The consumption of foods contaminated with AFB1 is also high in these regions, as exposure to mycotoxin biomarkers during pregnancy was observed in most prenatal exposure studies (Table 3). High levels of toxins are sometimes detected, but have not always been associated with deleterious effects on infant health. On the other hand, there are still no data directly linking the presence of this toxin with intrauterine or neonatal deaths in humans. Table 3 demonstrates the exposure to AFB1 during pregnancy by means of biomarkers.

In addition to the mechanisms of toxicity already cited in this review, studies indicate that anemia may be associated with exposure to AFs in humans [45]. Anemia can trigger several mechanisms that involve the activation of pro-inflammatory cytokines, and decreased amount of insulin-like growth hormones, which, in turn, leads to deleterious effects and prevents placental and fetal growth, and even fetal death [46].

**Table 3:** Biomarkers of aflatoxin B1 (AFB1) exposure found in pregnant women.

Period of Gestation	Biomarker	Country	Main Effects in Babies	Reference
1st Trimester	AFB1-Lisin (Serum)	Uganda	Low birth weight and smaller head circumference	[47]
1st–3rd Trimester	AF-Albumin (Serum)	Gambia	No data	[48]
3rd Trimester	AF-albumin (Serum) AFM1 (Urine)	Egypt	No data	[49]
2nd Trimester	AFM1 (Urine)	Zimbabwe	No data	[50]
No data	AFM1 (Urine)	China	No data	[51]
1st–2nd Trimester	AF-Albumin (Serum)	Gambia	DNA methylation in white cells	[52]
No data	AFB1-Lisin (Serum)	Ghana	Low birth weight	[53]
1st–2nd Trimester	AFB1-Lisin (Serum)	Tanzania	Small reduction in gestational age at delivery	[54]
1st–2nd Trimester	AFB1-Lisin (Serum)	Nepal	Babies small for gestational age	[55]

Populations living in developing countries, especially in Asia and Africa, are at higher risk of exposure to these substances, especially the most vulnerable populations and residents of rural areas that are impacted by drought and food insecurity. The lack of food may increase the consumption of grains that may often be improperly stored and may promote the intake of large quantities of mycotoxins [56,57,58,59].

In spite of the association between AFB1 biomarkers and negative effects on infants, aflatoxin should not be the only factor to be considered, as these populations are predisposed to the consumption of other mycotoxins, such as fumonisins. The association of this and other mycotoxins is a factor to be analyzed in future studies [60]. In addition, other factors such as maternal malnutrition and lack of prenatal care can have an impact on infant health. However, Lauer et al. [47] and Castelino et al. [48] evidenced an increase in the number of biomarkers in the maternal serum of women in Uganda and Gambia, respectively. This increase can be explained by the higher maternal metabolization during pregnancy, reflected in the greater production of enzymes of the cytochrome P450 family, which are essential in the metabolization of AFB1, and increase its toxicity. These factors, together with the changes evidenced in experimental studies, raise the hypothesis of an increased toxic potential of AFB1 in the prenatal period [18,19,20,25,26,61].

The Codex establishes up to 15 μg/kg of total aflatoxin food depending on the type of food, and in relation to AFB1, this limit is 5μg/kg of food [62]. The experiments in general use higher doses, but Suprya and Reddy, using a significantly lower dose compared to the others (10 μg/kg animal), showed lesions in the reproductive system of rats, which can serve as a warning sign in relation to safe doses of these substances during the prenatal period [30]. Furthermore, in general, the limits of aflatoxins in animal feed products are higher. In Brazil currently, the maximum limit of total aflatoxins for feed ingredients intended for animals is 50 μg/kg [63]. Therefore, studies aimed at standardizing doses and correlating them with effects in the prenatal period are important from the point of view of public health and may serve as a basis for changes in current legislation.

To elucidate the real impacts of the exposure to AFB1 on infant health, especially in the most susceptible populations, more studies must be conducted to determine safe intake during pregnancy and to suggest bases for legislation and awareness campaigns that can ensure acceptable consumption levels of mycotoxins.

## 5. Conclusions

Based on the data collected in this review, it can be concluded that AFB1 causes major impacts during embryonic and fetal development, which are the periods when animals are most susceptible to the effects of this mycotoxin. Among the effects reported are bone and visceral malformations, lesions in kidneys and liver, impacts in reproductive capacity, as well as genotoxicity and mutagenicity. Although most studies conducted are experimental, they serve as a window to glimpse at the actual effects of these substances. More data are needed to validate the effects on different species, considering the variability in resistance to the toxic effects, as well as the effect of the association between two or more mycotoxins, to better understand the impacts in animal production and on the health of pets. There are few studies on the influence of mycotoxins on the gestational period in humans, especially in developed countries. This phenomenon may be related to the fact that most developed countries have a subtropical climate and achieve better control over food contamination by mycotoxins, as well as having a very low birth rate, hindering access to broader population studies during the gestational phase. With few studies on humans, due to the high rates of stillbirths, miscarriages, and other complications during pregnancy, as well as the increasing number of cancer cases, even among children, more robust investigations are necessary to understand the real role of aflatoxins in these mechanisms. Finally, the study of these substances permeates food safety, animal health, and human health, and is a crucial example of the already consecrated concept of one health, which is shown to be more important every day.

## Figures and Tables

**Table 1:** Studies showing the effects of exposure to aflatoxin B1 (AFB1) on bone development.

Species	AFB1 Dose/PD/FE	Effects on Bone Development	Reference
Mice	20 mg/Kg PD: 7th or 13th FE: Intraperitoneal	Hypoplasia of the axial skeleton and metacarpal/metatarsal phalanges, cervical and coccygeal vertebrae. Failure in the ossification of the supraoccipital bone, pelvic and thoracic limbs.	[18]
Rabbits	0.05 mg/kg/day PD: 6th–18th FE: Gavage	Sternal and rib malformations. Failure in ossification of the skull, spine, vertebrae and ribs, carpus, tarsus, metatarsus, metacarpus, and phalanges. Decreased bone size in pelvic limb.	[19]
Rats	1 mg/kg PD: 6th–15th FE: Gavage	Failure in ossification of skull, thoracic and pelvic limbs, and spine. Change in shape and size of vertebrae. Absence of or decreased intervertebral disc size, incomplete formation of the pulposal nucleus, alteration and absence of bone accidents in limbs.	[20]

PD = Pregnancy Day. FE = Form of exposure.

**Table 2:** Studies showing the effects of exposure to aflatoxin B1 (AFB1) on organs.

Species	AFB1 Dose/PD/FE	Effects on Organs	Reference
Rabbits	0.05 mg/kg/day PD: 6th–18th FE: Gavage	Reduction in weight and absolute size of the viscera. Decreased size of the heart and ventricular lumen. Liver and kidneys containing vacuoles and congestion. Atrophy, glomerular degeneration, and disorganization of hepatocytes.	[19]
Rats	1 mg/kg PD: 6th–15th FE: Gavage	Hepatocyte degeneration and alteration of liver architecture. Congestion of the centrolobular vein and sinusoid capillaries. Kidneys presented tubular degeneration. Thymus presenting lymphoid depletion and reduction in epithelial differentiation.	[25]
Rats	10 μg/kg PD: 12th–19th FE: Intramuscular	Moderate degeneration of the testicles.	[26]
	20 μg/kg	Severe atrophy and reduction of germ cells of seminiferous tubules; reduced liver weight.	
	50 μg/kg	Severe degeneration, cell depletion, and epithelial rupture.	

PD = Pregnancy Day. FE = Form of exposure.

## Data Availability

Not applicable.

## References

[1] Eskola M., Kos G., Elliott C.T., Hajšlová J., Mayar S., Krska R. (2020). Worldwide contamination of food-crops with mycotoxins: Validity of the widely cited ‘FAO estimate’ of 25. Crit. Rev. Food Sci. Nutr..

[2] Bennett J.W., Klich M. (2003). Mycotoxins. Clin. Microbiol. Rev..

[3] Batatinha M.J., Simas M.M.S., Górniak S.L., Spinosa H.S., Górniak S.L., Palermo-Neto J. (2008). Micotoxicoses. Toxicologia Aplicada à Medicina Veterinária.

[4] Bando E., Gonçales L., Tamura N., Junior M. (2007). Biomarcadores para avaliação da exposição humana às micotoxinas. J. Bras. Patol. Med. Lab..

[5] Partanen H.A., El-Nezami H.S., Leppänen J.M., Myllynen P.K., Woodhouse H.J., Vähäkangas K.H. (2009). Aflatoxin B1 transfer and metabolism in human placenta. Toxicol. Sci..

[6] Khlangwiset P., Shephard G.S., Wu F. (2011). Aflatoxins and growth impairment: A review. Crit. Rev. Toxicol..

[7] Dhakal A., Sbar E. (2020). Aflatoxin toxicity. StatPearls.

[8] Mitchell N.J., Kumi J., Johnson N.M., Dotse E., Marroquin-Cardona A., Wang J.S., Jolly P.E., Ankrah N.A., Phillips T.D. (2013). Reduction in the urinary aflatoxin M1 biomarker as an early indicator of the efficacy of dietary interventions to reduce exposure to Aflatoxins. Biomarkers.

[9] Wild C., Turner P. (2002). The toxicology of Aflatoxins as a basis for public health decisions. Mutagenesis.

[10] Bujons J., Hsieh D.P., Kado N.Y., Messeguer A. (1995). Aflatoxin M1 8,9-epoxide: Preparation and mutagenic activity. Chem. Res. Toxicol..

[11] IARC (1993). IARC Monographs on the Evaluation of Carcinogenic Risks to Humans, Volume 56, Some Naturally Occurring Substances: Food Items and Constituents, Heterocyclic Aromatic Amines and Mycotoxins.

[12] IARC (2002). IARC Monographs on the Evaluation of Carcinogenic Risks to Humans, Volume 82, Some Traditional Herbal Medicines, Some Mycotoxins, Naphthalene and Styrene.

[13] Battacone G., Nudda A., Palomba M., Pascale M., Nicolussi P., Pulina G. (2005). Transfer of aflatoxin B1 from feed to milk and from milk to curd and whey in dairy sheep fed artificially contaminated concentrates. J. Dairy. Sci..

[14] Oliveira C.A.F., Sebastião L.S., Luciana S.S., Fagundes H., Rosim R.E., Fernandes A.M. (2010). Determinação de aflatoxina B1 em rações e aflatoxina M1 no leite de propriedades do Estado de São Paulo. Food Sci. Technol..

[15] Marchese S., Polo A., Ariano A., Velotto S., Costantini S., Severino L. (2018). Aflatoxin B1 and M1: Biological properties and their involvement in cancer development. Toxins.

[16] Giovati L., Magliani W., Ciociola T., Santinoli C., Conti S., Polonelli L. (2015). AFM1 in milk: Physical, biological, and prophylactic methods to mitigate contamination. Toxins.

[17] Benkerroum N. (2020). Chronic and acute toxicities of Aflatoxins: Mechanisms of action. Int. J. Environ. Res. Public Health.

[18] Abdulrazzaq Y., Padmanabhan R., Salim M., Kochyil J., Shafiullah M. (2011). Teratogenic effects of aflatoxin B1 in mice exposed in early and late gestation. Pediatr. Res..

[19] El-Nahla S.M., Imam H.M., Moussa E., Ibrahim A., Ghanam A. (2013). Teratogenic effects of aflatoxin in rabbits (*Oryctolagus cuniculus*). J. Vet. Anat..

[20] Fetaih H.A., Dessouki A.A., Hassanin A.A., Tahan A.S. (2014). Toxopathological and cytogenetic effects of aflatoxin B1 (AFB1) on pregnant rats. Pathol. Res. Pract..

[21] Chu F.S., Caballero B. (2003). Mycotoxins toxicology. Encyclopedia of Food Scienses and Nutrition.

[22] Hayes A.W., Hayes A.E. (1981). Aflatoxins. Mycotoxin Teratogenicity and Mutagenicity.

[23] Wangikar P., Dwivedi P., Sharma A., Sinha N. (2004). Effect in rats of simultaneous prenatal exposure to ochratoxin A and aflatoxin B1. II. Histopathological features of teratological anomalies induced in fetuses. Birth Defects Res. B Dev. Reprod. Toxicol..

[24] Wangikar P.B., Dwivedi P., Sinha N., Sharma A.K., Telang A.G. (2005). Teratogenic effects in rabbits of simultaneous exposure to ochratoxin A and aflatoxin B1 with special reference to microscopic effects. Toxicology.

[25] Fetaih H., Dessouki A., Tahan A. (2015). Toxopathological effect of AFB1 on pregnant rats. Global Anim. Sci. J..

[26] Supriya C., Reddy P.S. (2015). Prenatal exposure to aflatoxin B1: Developmental, behavioral, and reproductive alterations in male rats. Naturwissenschaften.

[27] Meissonnier G.M., Pinton P., Laffitte J., Cossalter A.M., Gong Y.Y., Wild C.P., Bertin G., Galtier P., Oswald I.P. (2008). Immunotoxicity of aflatoxin B1: Impairment of the cell-mediated response to vaccine antigen and modulation of cytokine expression. Toxicol. Appl. Pharmacol..

[28] Bastaki S.A., Osman N., Kochiyil J., Shafiullah M., Padmanabhan R., Abdulrazzaq Y.M. (2010). Toxicokinetics of aflatoxin in pregnant mice. Int. J. Toxicol..

[29] Hasanzadeh S., Hosseini E., Rezazadeh L. (2011). Effects of aflatoxin B1 on profiles of gonadotropic (FSH and LH), steroid (testosterone and 17β-estradiol) and prolactin hormones in adult male rat. Iran J. Vet. Res..

[30] Supriya C., Girish B.P., Reddy P.S. (2014). Aflatoxin B1-induced reproductive toxicity in male rats: Possible mechanism of action. Int. J. Toxicol..

[31] Fauzi A., Ahmed S., Abead S. (2015). Toxicity effect of aflatoxin B1 on reproductive system of albino male rats. Pak. J. Biol. Sci..

[32] Hasanzadeh S., Amani S. (2012). Aflatoxin B1 effects on ovarian follicular growth and atresia in the rat. Comp. Clin. Pathol..

[33] Althnaian T., Albokhadai I., El-Bahr S. (2016). Effect of aflatoxin B1 on histopathology and oxidative stress biomarkers in testis of rats with special references to gene expression of antioxidant enzymes. Int. J. Pharmacol..

[34] Abdel-Aziem S., Saleh Z., Farrag A. (2007). Impact of whey protein on the genotoxic effects of aflatoxins in rats. Int. J. Dairy Sci..

[35] Woo L.L., Egner P.A., Belanger C.L., Wattanawaraporn R., Trudel L.J., Croy R.G., Groopman J.D., Essigmann J.M., Wogan G.N., Bouhenguel J.T. (2011). Aflatoxin B1-DNA adduct formation and mutagenicity in livers of neonatal male and female B6C3F1 mice. Toxicol. Sci..

[36] Chawanthayatham S., Thiantanawat A., Egner P.A., Groopman J.D., Wogan G.N., Croy R.G., Essigmann J.M. (2014). Prenatal exposure of mice to the human liver carcinogen aflatoxin B1 reveals a critical window of susceptibility to genetic change. Int. J. Cancer.

[37] El-Bahr S.M. (2015). Effect of Curcumin on hepatic antioxidant enzymes activities and gene expressions in rats intoxicated with aflatoxin B1. Phytother. Res..

[38] Saad-Hussein A., Moubarz G., Mohgah S.A., Wafaa G.S., Aya H.M. (2019). Role of antioxidant supplementation in oxidant/antioxidant status and hepatotoxic effects due to aflatoxin B1 in wheat miller workers. J. Complement. Integr. Med..

[39] Dai Y., Huang K., Zhang B., Zhu L., Xu W. (2017). Aflatoxin B1-induced epigenetic alterations: An overview. Food Chem. Toxicol..

[40] Veselý D., Veselá D., Jelínek R. (1983). Comparative assessment of the aflatoxin B1, B2, G1, G2 and M1 embryotoxicity in the chick embryo. Toxicol. Lett..

[41] Sur E., Celik I. (2003). Effects of aflatoxin B1 on the development of the bursa of Fabricius and blood lymphocyte acid phosphatase of the chicken. Br. Poult. Sci..

[42] Wu T.S., Cheng Y.C., Chen P.J., Huang Y.T., Yu F.Y., Liu B.H. (2019). Exposure to aflatoxin B1 interferes with locomotion and neural development in zebrafish embryos and larvae. Chemosphere.

[43] Cheng Y.C., Wu T.S., Huang Y.T., Chang Y., Yang J.J., Yu F.Y., Liu B.H. (2021). Aflatoxin B1 interferes with embryonic liver development: Involvement of p53 signaling and apoptosis in zebrafish. Toxicology.

[44] Lawn J.E., Blencowe H., Waiswa P., Amouzou A., Mathers C., Hogan D., Flenady V., Frøen J.F., Qureshi Z.U., Calderwood C. (2016). Stillbirths: Rates, risk factors, and acceleration towards 2030. Lancet.

[45] Shuaib F.M., Jolly P.E., Ehiri J.E., Jiang Y., Ellis W.O., Stiles J.K., Yatich N.J., Funkhouser E., Person S.D., Wilson C. (2010). Association between anemia and aflatoxin B1 biomarker levels among pregnant women in Kumasi, Ghana. Am. J. Trop. Med. Hyg..

[46] Smith L.E., Prendergast A.J., Turner P.C., Humphrey J.H., Stoltzfus R.J. (2017). Aflatoxin exposure during pregnancy, maternal anemia, and adverse birth outcomes. Am. J. Trop. Med. Hyg..

[47] Lauer J.M., Duggan C.P., Ausman L.M., Griffiths J.K., Webb P., Wang J.S., Xue K.S., Agaba E., Nshakira N., Ghosh S. (2019). Maternal Aflatoxin exposure during pregnancy and adverse birth outcomes in Uganda. Matern. Child. Nutr..

[48] Castelino J.M., Dominguez-Salas P., Routledge M.N., Prentice A.M., Moore S.E., Hennig B.J., Wild C.P., Gong Y.Y. (2014). Seasonal and gestation stage associated differences in aflatoxin exposure in pregnant Gambian women. Trop. Med. Int. Health.

[49] Piekkola S., Turner P.C., Abdel-Hamid M., Ezzat S., El-Daly M., El-Kafrawy S., Savchenko E., Poussa T., Woo J.C., Mykkänen H. (2012). Characterisation of Aflatoxin and D=deoxynivalenol exposure among pregnant Egyptian women. Food Addit. Contam..

[50] Smith L.E., Mbuya M., Prendergast A.J., Turner P.C., Ruboko S., Humphrey J.H., Nelson R.J., Chigumira A., Kembo G., Stoltzfus R.J. (2017). Determinants of recent aflatoxin exposure among pregnant women in rural Zimbabwe. Mol. Nutr. Food Res..

[51] Lei Y., Fang L., Akash M.S.H., Rehman K., Liu Z., Shi W., Chen S. (2013). Estimation of urinary concentration of aflatoxin M1 in Chinese pregnant women. J. Food. Sci..

[52] Hernandez-Vargas H., Castelino J., Silver M.J., Dominguez-Salas P., Cros M.P., Durand G., Calvez-Kelm F.L., Prentice A.M., Wild C.P., Moore S.E. (2015). Exposure to aflatoxin B1 in utero is associated with DNA methylation in white blood cells of infants in The Gambia. Int. J. Epidemiol..

[53] Shuaib F.M., Jolly P.E., Ehiri J.E., Yatich N., Jiang Y., Funkhouser E., Person S.D., Wilson C., Ellis W.O., Wang J.S. (2010). Association between birth outcomes and aflatoxin B1 biomarker blood levels in pregnant women in Kumasi, Ghana. Trop. Med. Int. Health.

[54] Passarelli S., Bromage S., Darling A.M., Wang J.S., Aboud S., Mugusi F., Griffiths J.K., Fawzi W. (2020). Aflatoxin exposure in utero and birth and growth outcomes in Tanzania. Mater. Child. Nutr..

[55] Andrews-Trevino J.Y., Webb P., Shively G., Rogers B., Baral K., Davis D., Paudel K., Pokharel A., Shrestha R., Wang J.S. (2020). Dietary determinants of aflatoxin B1-lysine adduct in pregnant women consuming a rice-dominated diet in Nepal. Eur. J. Clin. Nutr..

[56] Bryden W.L. (2007). Mycotoxins in the food chain: Human health implications. Asia Pac. J. Clin. Nutr..

[57] Wagacha J.M., Muthomi J.W. (2008). Mycotoxin problem in Africa: Current status, implications to food safety and health and possible management strategies. Int. J. Food Microbiol..

[58] Mohd-Redzwan S., Jamaluddin R., Abd-Mutalib M.S., Ahmad Z. (2013). A mini review on aflatoxin exposure in Malaysia: Past, present and future. Front. Microbiol..

[59] Zhang W., Liu Y., Liang B., Zhang Y., Zhong X., Luo X., Huang J., Wang Y., Cheng W., Chen K. (2020). Probabilistic risk assessment of dietary exposure to aflatoxin B1 in Guangzhou, China. Sci. Rep..

[60] Chen C., Riley R.T., Wu F. (2018). Dietary fumonisin and growth impairment in children and animals: A review. Food Sci. Food Saf..

[61] Tracy T.S., Venkataramanan R., Glover D.D., Caritis S.N. (2005). Temporal changes in drug metabolism (CYP1A2, CYP2D6 and CYP3A Activity) during pregnancy. Am. J. Obstet. Gynecol..

[62] Food and Agriculture Organization of the United Nations (FAO), World Health Organization Codex Alimentarius International Food Standards, General Standard for Contaminants and Toxins in Food and Feed, Codex Stan CXS 193 1995 (CxS_193-2015). https://www.fao.org/input/download/standards/17/CXS_193e_2015.pdf.

[63] MAPA (1988). Portaria MA/SNAD/SFA no 07, de 09 de Novembro de 1988. Diário Oficial da União, Poder Executivo, Brasília, DF, 9 de Nov. 1988. Seção 1, p. 21.968. https://www.jusbrasil.com.br/diarios/DOU/1988/11/09.

